# Natural Deep Eutectic Solvent Extraction Optimization of Alkaloids from *Mitragyna speciosa* : A Methodological Evaluation

**DOI:** 10.1055/a-2880-8498

**Published:** 2026-06-11

**Authors:** Christina Koufali, Alexandros Polyzois, Marina Kritsanida, Nektarios Aligiannis, Ioanna Chinou, Nikolas Fokialakis

**Affiliations:** 1 Division of Pharmacognosy & Chemistry of Natural Products, Department of Pharmacy, School of Health Sciences, National and Kapodistrian University of Athens, Athens, Greece; 2 UMR CNRS 8038 CiTCom, Faculté de Pharmacie, Université Paris Cité, Paris, France

**Keywords:** alkaloids, natural deep eutectic solvents (NaDES), *Mitragyna speciosa*, Rubiaceae, recovery

## Abstract

*Mitragyna speciosa* (kratom) is a psychoactive plant native to Southeastern Asia, belonging to the Rubiaceae family. Traditionally, its fresh leaves have been consumed in the region for centuries due to their therapeutic properties, including pain relief. The leaves contain a variety of phytochemicals with the alkaloids being the most biologically significant constituents. In the present study–the first investigation exploring sustainable extraction methods for kratom–47 natural deep eutectic solvent (NaDES) systems were screened. The most effective system demonstrated significantly higher extraction efficiency compared to commonly used organic solvents. The food-grade system, lactic acid : glucose was subsequently selected for optimization using a one-factor-at-a-time approach, and the optimal conditions were a molar ratio of 4 : 1 and a water content of 20%. Further optimization using a Box–Behnken design and response surface methodology identified the optimal extraction conditions as a solid-to-liquid ratio of 30 mg/mL, a temperature of 70 °C and an ultrasound treatment time of 12 min. Finally, three recovery methods were evaluated: solid phase extraction (including strong cation exchange and reversed-phase types), macroporous resins (six types), and acid-base extraction. The highest recovery yields were obtained with strong cation exchange solid phase extraction (95%) followed by the macroporous resins XAD 18 and XAD 1600 N (85%).

## Introduction

*Mitragyna speciosa* (Korth.) Havil., commonly known as ‘kratomʼ in Thailand, ‘ketumʼ or ‘biak-biakʼ in Malaysia, and ‘mambogʼ in the Philippines, is a psychoactive plant native to Southeastern Asia belonging to the Rubiaceae family 1 , 2 . Traditionally, the fresh leaves of *M. speciosa* have been consumed in Southeast Asia for their therapeutic properties for centuries. People consume raw kratom leaves by chewing them, brewing them into tea, or smoking them 2 , 3 . The leaves of kratom have been used to relieve pain, hypertension, coughs, and diarrhea, reduce opioid use and relieve withdrawal, and increase sexual desire 4 , 5 . Additionally, at low doses, it acts as a stimulant, while at higher doses, it produces opioid-like effects, causing sedation. It boosts stamina, enhances mood, and helps reduce anxiety, with its stimulating effects helping to increase energy and improve work productivity 1 , 5 . Kratom leaves contain a variety of phytochemicals such as alkaloids, triterpenes, and flavonoids 6 ; however, the literature focuses on the rich alkaloid content 1 , 7 , 8 .

Over 40 alkaloids have been isolated from leaves of *M. speciosa* mainly belonging to tetracyclic indole alkaloids and pentacyclic oxindole alkaloids 3 , 7 . Mitragynine is the primary alkaloid found in the leaves, along with its isomers such as speciogynine, speciociliatine, and mitraciliatine 9 , 10 . These compounds exhibit anti-inflammatory properties by suppressing prostaglandin E2 production by inhibiting cyclooxygenase-2 expression 11 . Studies also show that mitragynine-like alkaloids exhibit high affinity for serotonin receptors (5-HT1ARs and 5-HT2BRs) 12 . Additionally, mitragynine has been shown to block T- and L-type calcium channels, reducing calcium influx in neuronal cells, which suggests the potential to alter smooth muscle contraction dynamics 13 . Therefore, kratom alkaloids are considered important pharmaceutical agents.

Organic solvents such as methanol and chloroform are mostly used to extract alkaloids from *M. speciosa* 9 . Although effective, these solvents present major disadvantages including toxicity, flammability, poor biodegradability, and high cost 14 . This has driven interest in greener alternatives such as deep eutectic solvents (DES), which are mixtures of two or three components that form strong intermolecular interactions, primarily hydrogen bonding, along with Van der Waals and electrostatic forces. These interactions result in a eutectic mixture with a melting point much lower than that of its individual components 15 , 16 .

Natural deep eutectic solvents (NaDES) are mixtures of natural compounds such as amino acids, organic acids, sugars, and choline derivatives that form eutectic systems at specific molar ratios of hydrogen bond acceptors (HBA) and donors (HBD), resulting in a minimum melting point 17 , 18 . These solvents have attracted increasing interest due to their low toxicity, biodegradability, and renewable origin 16 , 19 , making them suitable for pharmaceutical, food, and cosmetic applications. Although mainly composed of hydrophilic metabolites, NaDES can also dissolve poorly water-soluble compounds 20 and have proven effective for extracting various bioactive molecules, including flavonoids 21 , anthraquinones 22 , curcuminoids 23 , alkaloids 24 , and saponins 25 .

This study to the best of our knowledge presents the first comparative investigation of the efficacy of hydrophilic NaDES to extract mitragynine-type alkaloids from *M. speciosa* leaves, together with the development of recovery techniques. The evaluation of 47 NaDES systems was performed using the quantitative analysis of alkaloid standards. Furthermore, a one-factor-at-a-time optimization of the most promising NaDES was carried out along with the statistical optimization of the extraction parameters using a Box–Behnken design through response surface methodology (RSM). Finally, three recovery techniques were investigated: solid phase extraction, macroporous resin, and acid base extraction.

## Results and Discussion

The primary objective of this study was to investigate the capacity of NaDES solvent systems to extract mitragynine-type alkaloids and to identify a system capable of extracting the target compounds as effectively as organic solvents. To achieve this, 47 NaDES solvent systems ( **Table 1S** , Supporting Information) were formulated from natural, environmentally friendly low-toxicity food-grade components. The formulated NaDES were classified into five categories based on HBA or HBD: choline-based, betaine-based, proline-based, glycerol-based, and acid/sugar-based NaDES.

Prior to the extraction process, viscosity and pH values of the tailor-made solvents were investigated. The physicochemical properties of NaDES including viscosity and pH can significantly influence the extraction efficiency of the system 26 . Viscosity is a major limiting factor of NaDES, as it restricts the diffusion of secondary metabolites within the solvent matrix. To overcome this limitation, water is commonly added during NaDES preparation.

The addition of water decreases viscosity and improves diffusion into plant material 24 . However, when the water content exceeds 50% (v/v), it can disrupt the hydrogen-bonding network of the NaDES system 15 , 25 . Consequently, during the screening process, 30% water was added to each NaDES formulation.

The pH value is also a factor that affects extraction efficiency in a NaDES solvent system, as it determines the solubility of secondary metabolites. In the case of alkaloids, acidic conditions favor protonation of the nitrogen atom, leading to the formation of alkaloid salts that are highly soluble in polar solvents. In contrast, under alkaline conditions, alkaloids are deprotonated to their free base form, which exhibits greater solubility in non-polar solvents. Thus, tuning the pH of NaDES systems is essential for optimizing alkaloid recovery and selectivity.

In our study, we evaluated the correlation between extraction efficiency with viscosity and pH using Spearmanʼs rank correlation coefficient. A negative, moderate correlation ( *ρ* = − 0.32, p = 0.34) was observed between pH and the total alkaloid content ( Fig. 1 a ), indicating that lower pH values could be associated with higher alkaloid yield. This effect can be explained by the increased solubility of alkaloids in acidic polar solvent systems. Furthermore, a negative strong correlation ( *ρ* = − 0.69, p < 0.001) was found between viscosity and the total alkaloid content ( Fig. 1 b ), demonstrating that high viscosity significantly reduces extraction efficiency. Overall, these findings highlight that primarily viscosity and secondarily pH play important roles in the efficiency of a NaDES extraction.

**Fig. 1:**
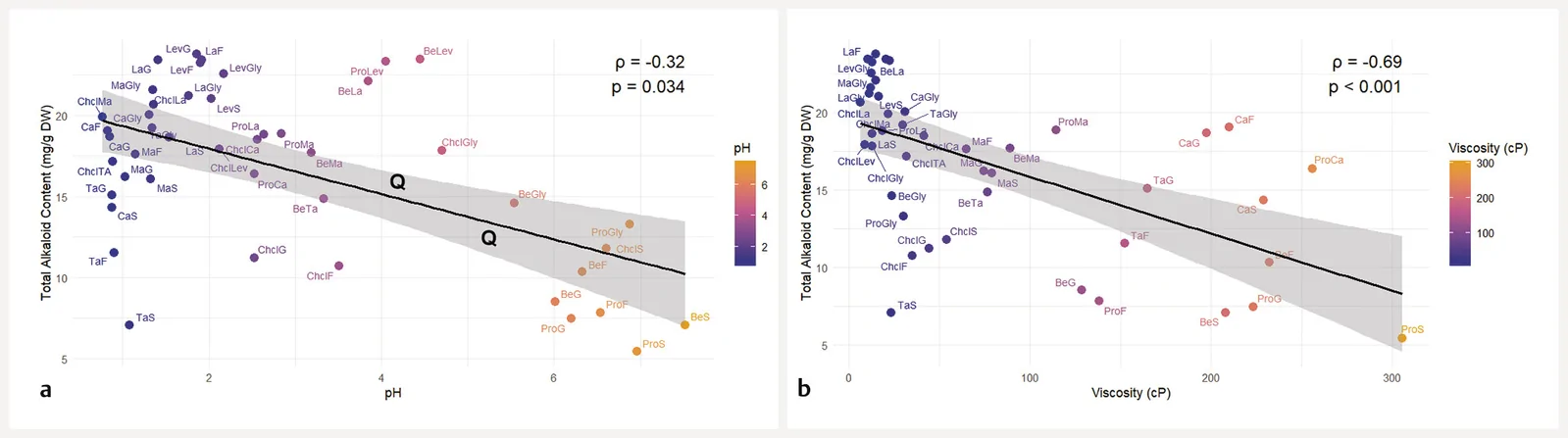
Correlation of the total alkaloid content with **a** pH and **b** viscosity determined by Spearmanʼs rank correlation coefficient ( *ρ* ) rho.

The content of mitragynine, speciogynine, speciociliatine ( Fig. 2 ), and total alkaloid content were quantified, while mitraphylline and 7-hydroxy-mitragynine ( Fig. 2 ), though included in the standards, were not detected in the plant material. The results are presented according to NaDES category: choline-based NaDES, betaine-based NaDES, proline-based NaDES, glycerol-based NaDES, and acid/sugar-based NaDES ( **Fig. 1S-4S** , Supporting Information). Each category showed significant variations (p < 0.05) in extraction efficiency. Commonly used conventional solvents were also evaluated ( **Fig. 5S** , Supporting Information), and methanol, which showed the highest extraction efficiency among them, was selected as the reference solvent for comparison with the most efficient NaDES systems ( Fig. 3 ). The maximum total alkaloid content was observed in BeLev 1 : 1 (23.8 ± 0.3 mg/g), LaG 2 : 1 (23.4 ± 0.2 mg/g), LevF 2 : 1 (23.5 ± 0.3 mg/g), LevG 2 : 1 (23.9 ± 0.3 mg/g), LevGly 1 : 2 (23.2 ± 0.3 mg/g), and ProLev 1 : 1 (23.7 ± 0.1 mg/g), which did not differ significantly from each other. A slightly lower yield was observed for LaF 2 : 1 (22.5 ± 0.6 mg/g), while BeLa 1 : 1 (21.5 ± 0.4 mg/g) and MaGly (21.2 ± 0.3 mg/g) showed significantly lower contents. Among all the efficient NaDES tested and methanol, methanol exhibited the lowest total alkaloid content (20.7 ± 0.2 mg/g). This finding further highlights the superior extraction efficiency of NaDES compared to traditional organic solvents.

**Fig. 2:**
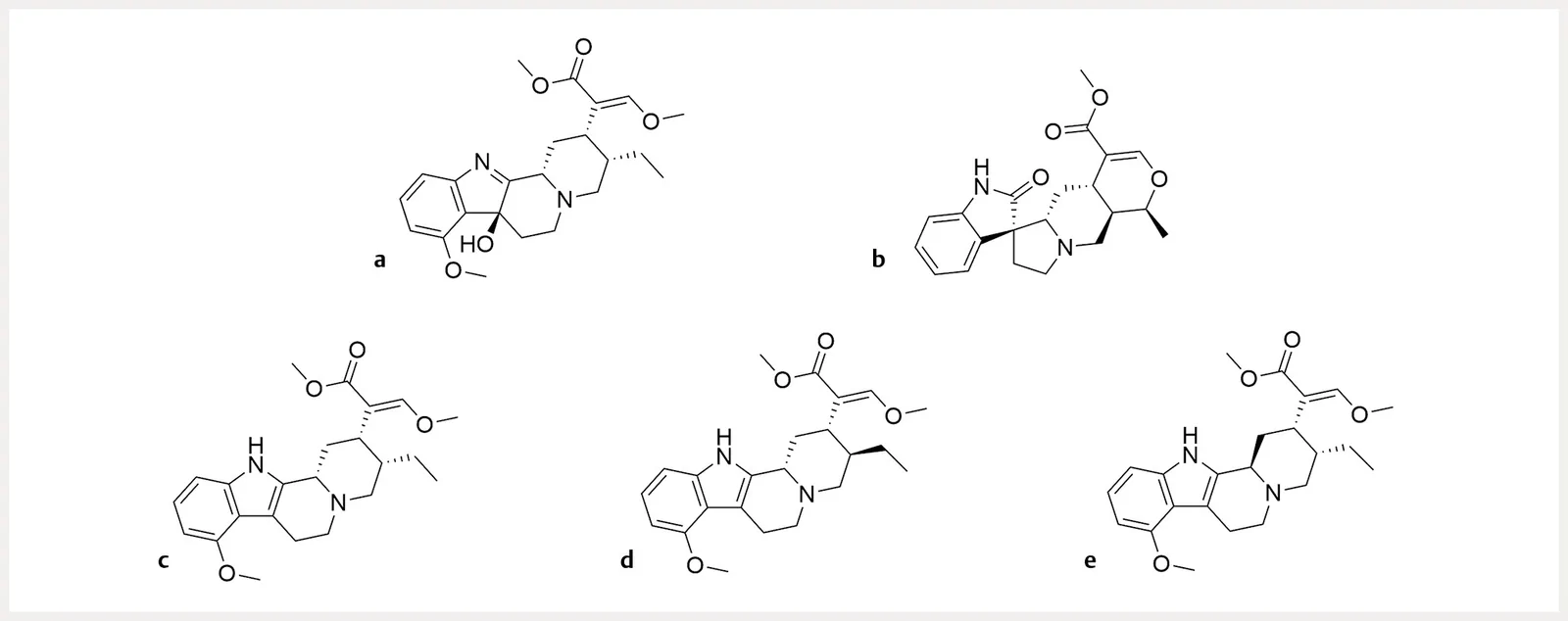
Chemical structures of **a** 7-hydroxy-mitragynine, **b** mitraphylline, **c** mitragynine, **d** speciociliatine and **e** speciogynine.

**Fig. 3:**
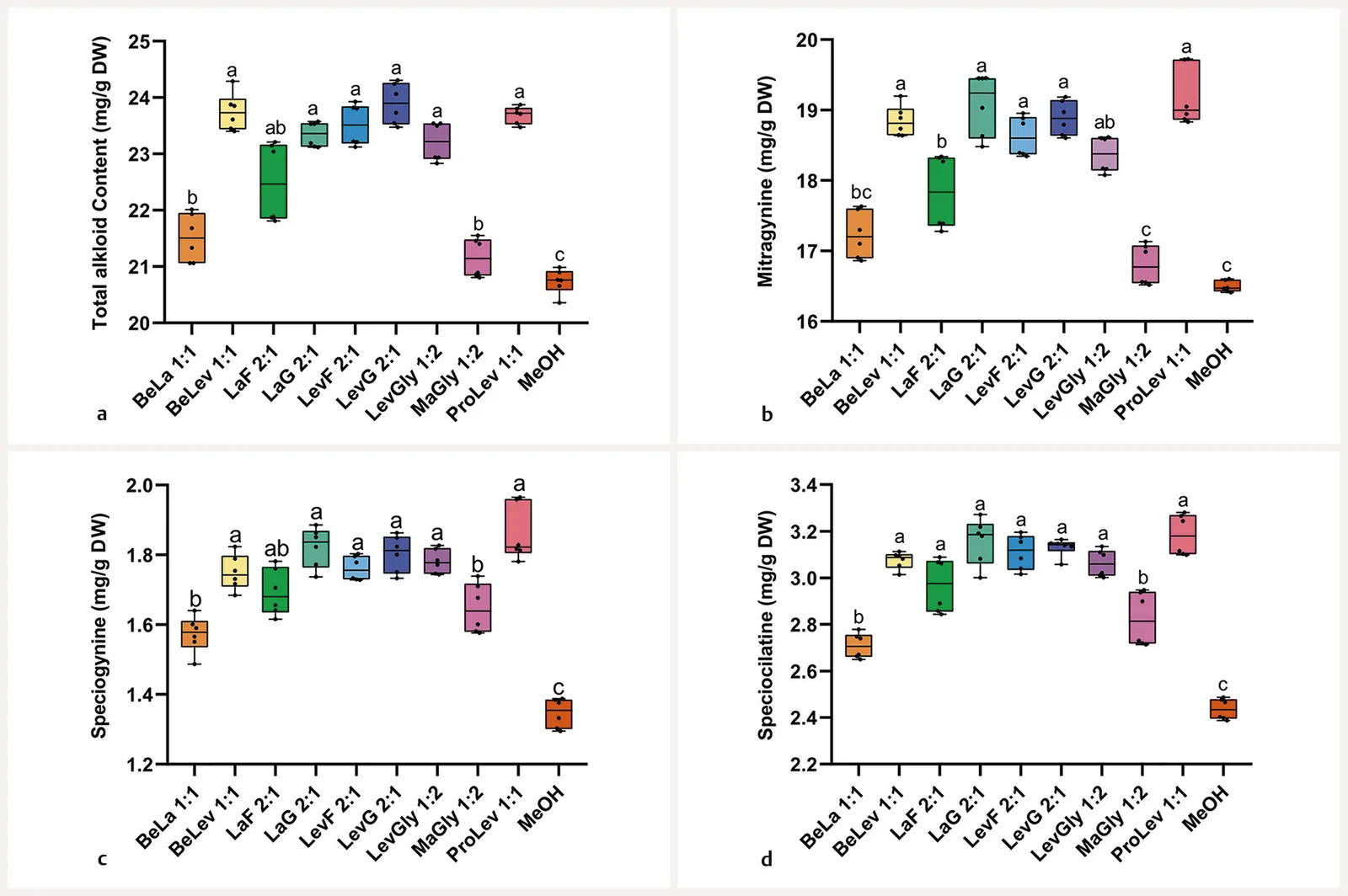
Evaluation of the most efficient natural deep eutectic solvents NaDES compared to conventional solvents. Yields are shown for **a** the total alkaloid content, **b** mitragynine, **c** speciogynine and **d** speciociliatine. Data are presented as mean ± SD (n = 6). Statistical significance was determined using Tukeyʼs test (p < 0.001). Different letters showed significant differences at p < 0.05. Boxes represent the interquartile range (25th–75th percentiles) with the median indicated by a line. Whiskers extend from the minimum to the maximum values. All individual data points are shown.

The solvent systems BeLev 1 : 1, LaG 2 : 1, LevF 2 : 1, LevG 2 : 1, LevGly 1 : 2, and ProLev 1 : 1 were identified as the most promising candidates for further investigation, as they yielded the highest total alkaloid contents, measured at 23.8 ± 0.3 mg/g, 23.4 ± 0.2 mg/g, 23.5 ± 0.3 mg/g, 23.9 ± 0.3 mg/g, 23.2 ± 0.3 mg/g, and 23.7 ± 0.1 mg/g, respectively. When selecting a final NaDES system for development, additional criteria relevant to large-scale industrial applications such as stability, cost-effectiveness, and regulatory acceptability were also considered. Based on these factors, LaG 2 : 1 was selected for further optimization, as it not only exhibited one of the highest alkaloid yields but also offered better economic and practical advantages for scale-up. Moreover, both lactic acid and glucose are food-grade ingredients with well-established safety profiles, further supporting the suitability of this system for food and nutraceutical applications.

Based on screening process, LaG 2 : 1 was selected for further investigation, as it was one of the most promising NaDES systems for mitragynine-type alkaloid extraction. To optimize this system, various molar ratios of HBA HBD (1 : 1 – 1 : 6) were evaluated for their extraction efficiency ( Fig. 4 ).

**Fig. 4:**
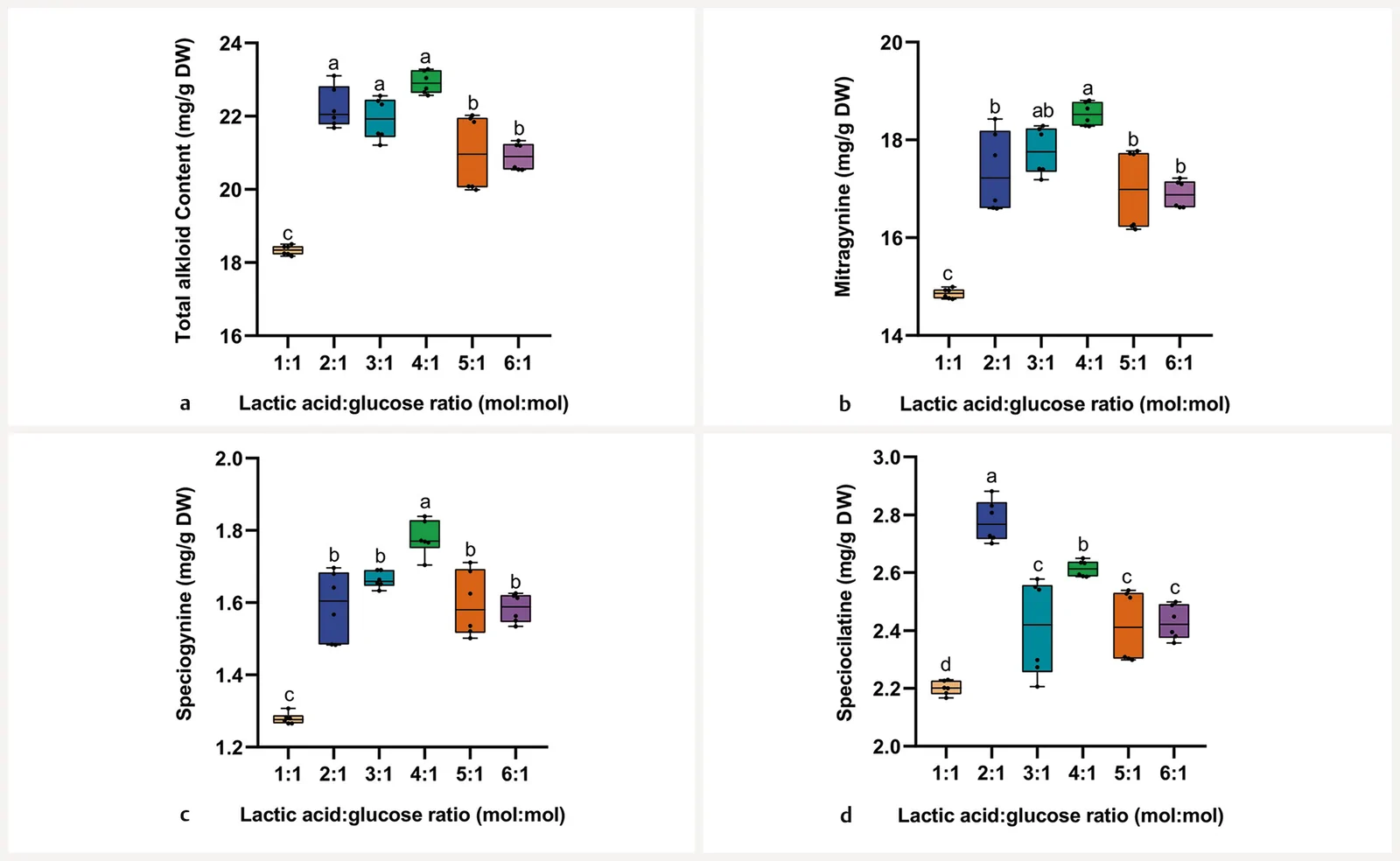
Effect of lactic acid : glucose (LaG) molar ratios on extraction efficiency (30% water, ultrasound-assisted extraction (UAE) 30 min, 40 °C). Yields are shown for **a** the total alkaloid content, **b** mitragynine, **c** speciogynine and **d** speciociliatine. Data are presented as mean ± SD (n = 6). Statistical significance was determined using Tukeyʼs test (p < 0.001). Different letters showed significant differences at p < 0.05. Boxes represent the interquartile range (25th–75th percentiles) with the median indicated by a line. Whiskers extend from the minimum to the maximum values. All individual data points are shown.

The results demonstrated that the different molar ratios significantly affected alkaloid yield. A sharp increase in alkaloid content was observed when the ratio increased from 1 : 1 to 2 : 1, indicating that higher lactic acid content enhances the solvating ability of the medium. Furthermore, the ratio of 2 : 1 to 4 : 1 produced the highest extraction yields, with no significant difference between them. In contrast, further increases to the ratio (5 : 1 and 6 : 1) reduced the extraction efficiency of the system. Considering these findings, the lactic acid : glucose system at a 4 : 1 molar ratio was selected for further optimization, as it demonstrated the best extraction efficiency.

Water content is a crucial factor influencing the extraction efficiency of NaDES. Based on the considerations described above, the water content of the selected NaDES system was further optimized by evaluating different levels (0%, 10%, 20%, 30%, and 40%). A gradual increase in alkaloid yield was observed from 0% to 20% water, whereas water content above 20% resulted in lower yields ( Fig. 5 ). Therefore, the addition of 20% water content was considered optimal for the LaG NaDES solvent system in the extraction of mitragynine-type alkaloid.

**Fig. 5:**
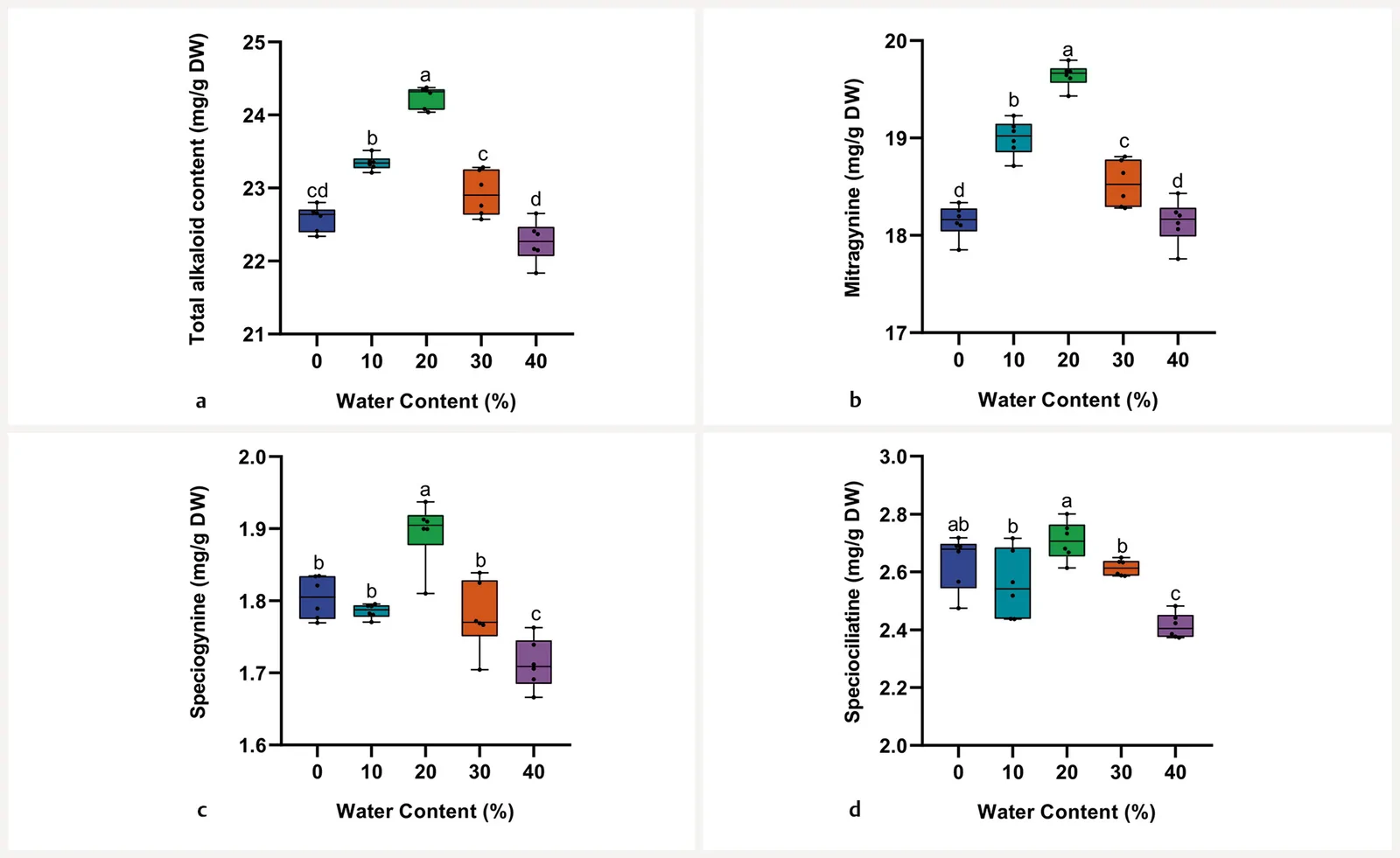
Effect of water content in the lactic acid : glucose (LaG) (4 : 1 mol : mol, ultrasound-assisted extraction (UAE) 30 min, 40 °C). Yields are shown for **a** the total alkaloid content, **b** mitragynine, **c** speciogynine and **d** speciociliatine. Data are presented as mean ± SD (n = 6). Statistical significance was determined using Tukeyʼs test (p < 0.001). Different letters showed significant differences at p < 0.05. Boxes represent the interquartile range (25th–75th percentiles) with the median indicated by a line. Whiskers extend from the minimum to the maximum values. All individual data points are shown.

To maximize extraction efficiency, the extraction parameters were optimized using a Box–Behnken design (BBD) with response surface methodology (RSM). Three independent variables at three levels used in the BBD design matrix include (i) extraction temperature (30, 50, and 70 °C), (ii) ultrasound time (10, 40, and 70 min), and (iii) liquid-to-solid ratio (30, 80, and 130 mg/mL). The full factorial BBD matrix involving 15 randomized experimental runs, coded at three levels (1, 0, and + 1) ( **Table 2S** , Supporting Information). The extraction efficiency of total alkaloid content was selected as the response to build a quadratic model by applying a second-order polynomial equation. The relevance of the model was evaluated using the squared correlation coefficient (R ^2^ ) and the lack-of-fit test by the analysis of variance (ANOVA) at a 95% confidence level. The model was significant (p < 0.001 and F = 64.04), while lack of fit was not significant, confirming that the experimental data were in good agreement with the predicted values. The results of ANOVA are summarized in **Table 3S** (Supporting Information).

The second-order polynomial quadric equation for total alkaloid content was obtained as follows:

*Y* = 25.92 + 0.3 *A* −0.1038 *B* −1.9888 *C* −0.2875 *AB* + 0.0875 *AC* −0.105 *BC* + 0.070 *A* ^2^ 0.0225 *B* ^2^ −0.4375 *C* ^2^

where Y represents the total alkaloid content (mg/g DW), A, extraction temperature (°C), B, ultrasound time (min), and C, solid-to-liquid ratio (mg/mL).

The 3D response surfaces and contour plots ( Fig. 6 ) illustrate the interactive effects of the independent variables on the extraction efficiency. Among the variables studied, the most influencing factor was the solid-to-liquid ratio (mg/mL) (p < 0.0001), followed by the extraction temperature (p < 0.05) and the ultrasound time (p < 0.3) that was not significant. According to the contour plots, alkaloid extraction increased significantly at the minimum solid-to-liquid ratio (30 mg/mL), with the optimal temperature in the range of 60 to 70 °C and ultrasound time of 10 – 20 min. This can be explained by the fact that higher temperatures enhance solubility and mass transfer, while prolonged sonication may cause degradation of the targeted compounds.

**Fig. 6:**
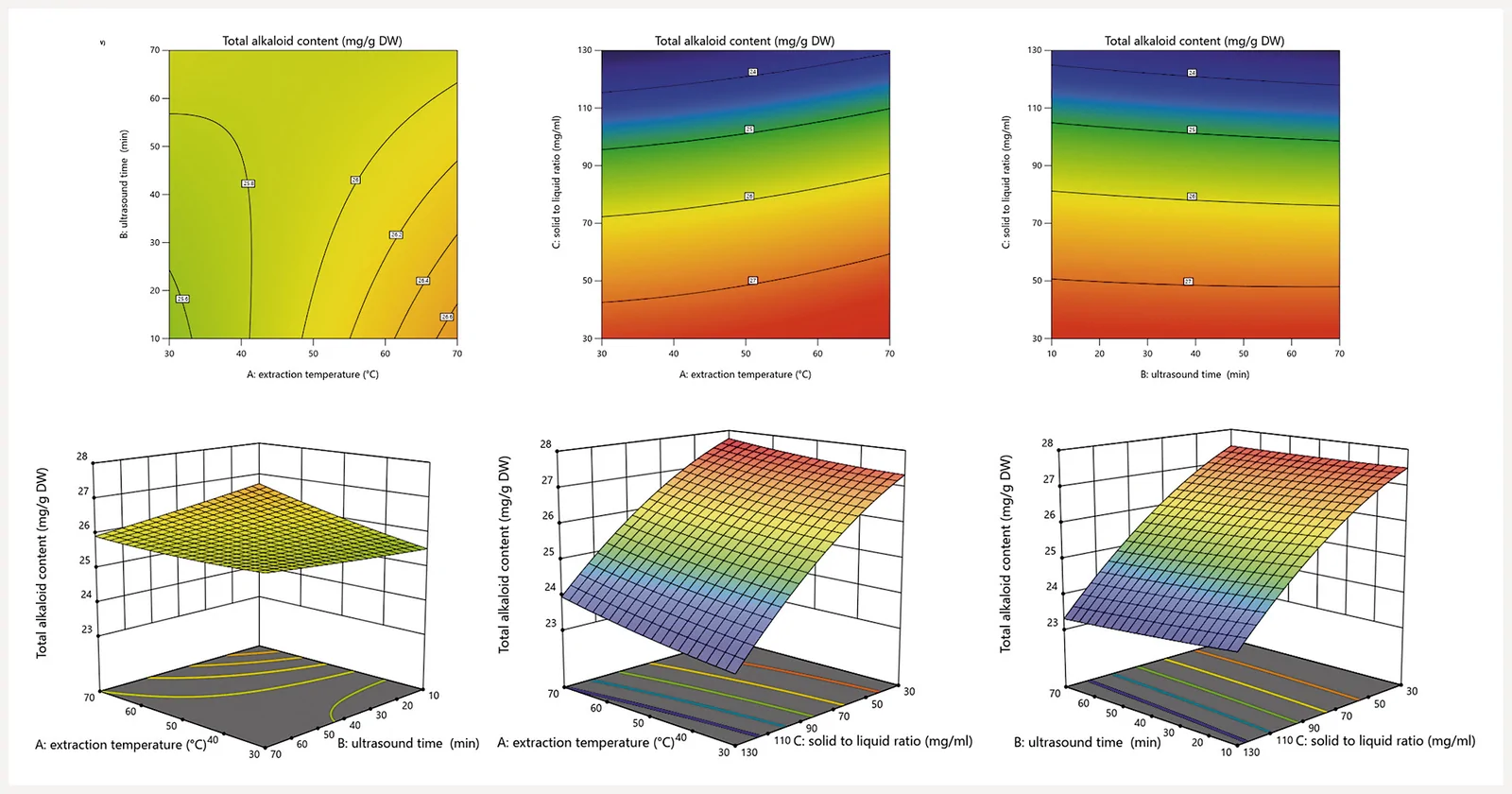
Response surface and contour for total alkaloid content (mg/g DW) in relation to **a** extraction time (min), **b** extraction temperature (°C), **c** solid-to-liquid ratio (mg/mL).

Based on the 3D response surfaces and contour plots, the optimal conditions for mitragynine-type alkaloid extraction were a solid-to-liquid ratio at 30 mg/mL, a temperature of 70 °C, and ultrasound time of 12 min. Triplicate experiments were conducted under these conditions, and the mean values were compared with the predicted values. The experimental extraction efficiencies fell within the 95% prediction interval (PI), confirming that the developed quadratic models are statistically reliable and reasonable. Therefore, the combination of ~ 70 °C, 12 min, and 30 mg/mL was identified as the optimal set of parameters for achieving maximum alkaloid content. Notably, the exceptionally short extraction time of 12 minutes highlights the high efficiency of this protocol, offering a rapid and time-effective approach compared to traditional extraction methods.

Recovery methods are necessary for bioactive compounds from NaDES extracts due to the solventʼs high viscosity and low vapor pressure, which are the main disadvantages of NaDES 16 , 27 . In the present study, three different techniques were evaluated: solid-phase extraction, which is a very common and useful method for laboratory-scale applications; macroporous resins, which can be applied at an industrial scale; and acid-base extraction, which is advantageous due to its minimal use of consumables.

Solid-phase extraction is a common and well-established technique for purifying natural compounds from complex mixtures. In this study, reversed phase (C18) and strong cation exchange (SCX) cartridges were selected for alkaloids recovery. C18 cartridges are widely used for secondary metabolites and have previously been applied for alkaloid separation from *M. speciosa* 28 , and SCX cartridges are commonly employed for alkaloid enrichment 29 .

The recovery using SPE cartridges was efficient and straightforward. The NaDES solvent system was successfully removed, and the recovery of the target compounds was satisfactory in both cases ( Fig. 7 ). The recovery yields (%) for mitragynine, speciogynine, speciociliatine, and total alkaloid content were 97%, 91%, 83%, and 95%, respectively, for SCX SPE and 91%, 86% 89%, and 91% for C18 SPE. These results demonstrate that SPE is an effective technique for NaDES recovery. SCX cartridges provided better recovery results than C18, probably because mitragynine-type alkaloids are basic nitrogen compounds that become positively charged under acidic conditions and are retained on the cartridge through strong ionic interactions. In contrast, C18 rely on hydrophobic interactions, which are less specific for these targeted compounds.

**Fig. 7:**
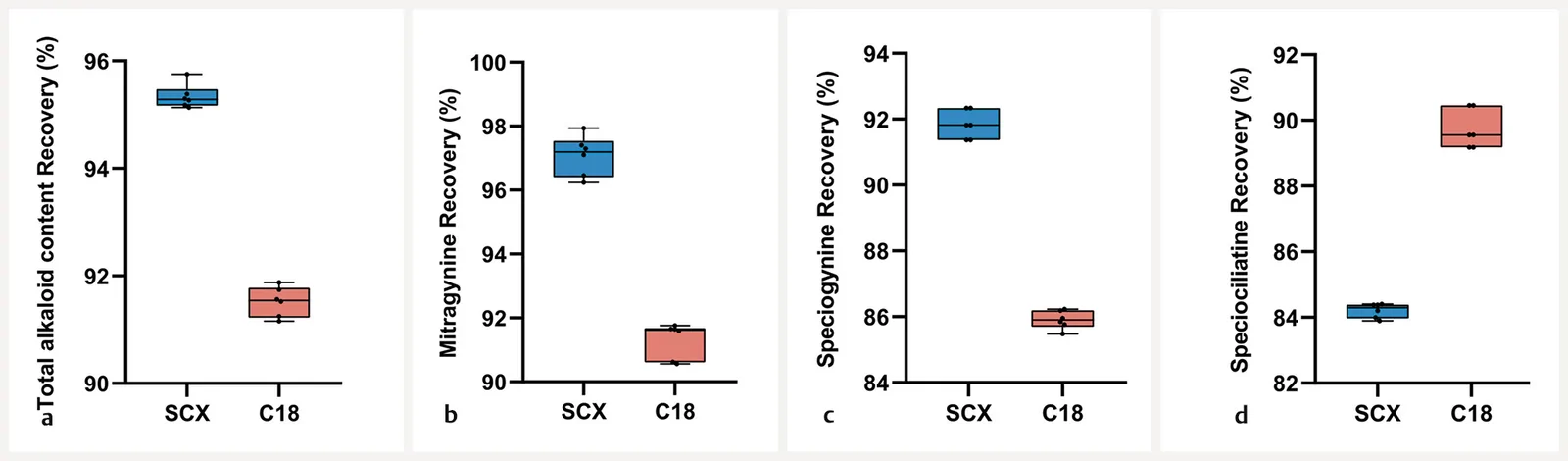
Recovery of mitragynine-type alkaloids from natural deep eutectic solvents (NaDES) extract by solid-phase extraction. Data are presented on % of recovery, n = 6, p < 0.001. Boxes represent the interquartile range (25th–75th percentiles) with the median indicated by a line. Whiskers extend from the minimum to the maximum values. All individual data points are shown.

Macroporous resins have been widely employed for NaDES recovery 14 , 30 and extensively in recovering bioactive compounds at industrial scale from food and food waste 31 . In this present study, six different resins were evaluated for mitragynine-type alkaloids recovery: Amberlite XAD-4, Amberlite XAD-HD7, Amberlite XAD-16 N, Amberlite XAD-1600 N, Amberlite XAD-1180 N, and Amberlite XAD-18 ( Fig. 8 ).

**Fig. 8:**
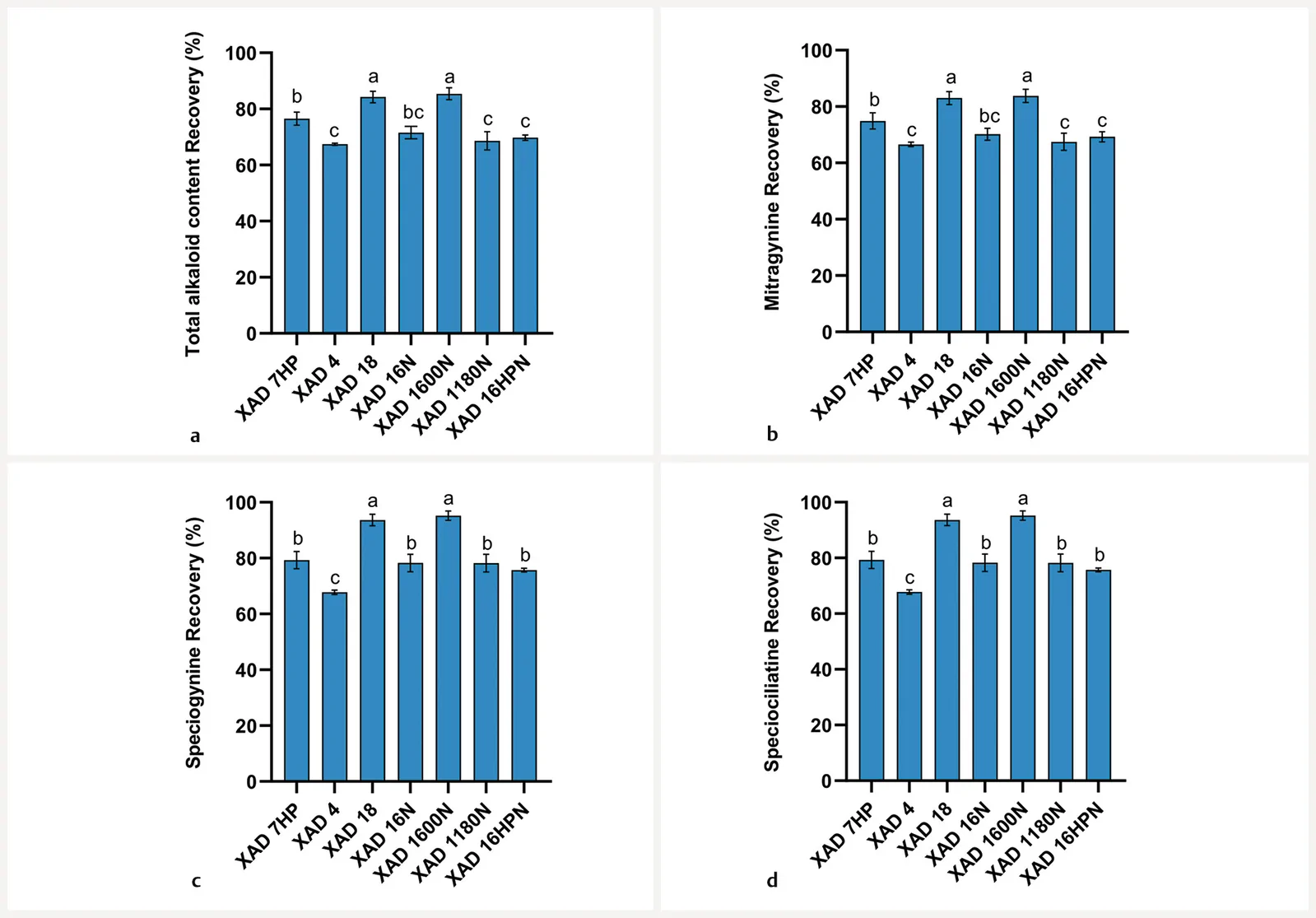
Recovery of mitragynine-type alkaloids from natural deep eutectic solvents (NaDES) extract by macroporous resins. Data are presented as mean ± SD (n = 6). Statistical significance was determined using Tukeyʼs test (p < 0.001). Different letters showed significant differences at p < 0.05.

Among them, resins Amberlite XAD 18 and Amberlite XAD 1600 N exhibited the highest yields, achieving recoveries of 83%, 93%, 88%, and 85% for mitragynine, speciogynine, speciociliatine, and total alkaloid content, respectively. This performance can be attributed to their similar particle size, surface area, and pore diameter, as well as their styrene–divinylbenzene matrix that favors hydrophobic and *π* - *π* interactions.

Interestingly, XAD-7HP also showed good recovery yields despite its more polar aliphatic backbone. These results may be explained because of the acid environment of the NaDES system, which converts alkaloids in their protonated salt form, thereby increasing their polarity and affinity for the more polar acrylic resin. The high recovery rates of Amberlite XAD-18 and Amberlite XAD-1600 N, together with the reusability of these resins, suggest a sustainable and efficient method for recovering these targeted compounds from NaDES extract for large-scale applications.

Acid-base extraction is a common method for alkaloid enrichment and has previously been applied to mitragynine-type alkaloids 32 . To the best of our knowledge, this study is the first to employ this method for the recovery of alkaloids from a NaDES extract.

The recovery of alkaloids from the NaDES extract using acid-base extraction was satisfactory for all target compounds ( Fig. 9 ). The recoveries were 65%, 85%, 66%, and 69% for mitragynine, speciogynine, speciociliatine, and total alkaloid content, respectively. Although these values were lower than those obtained with SPE and macroporous resins, acid-base extraction may still offer advantages due its simplicity and minimal requirement for consumables.

**Fig. 9:**
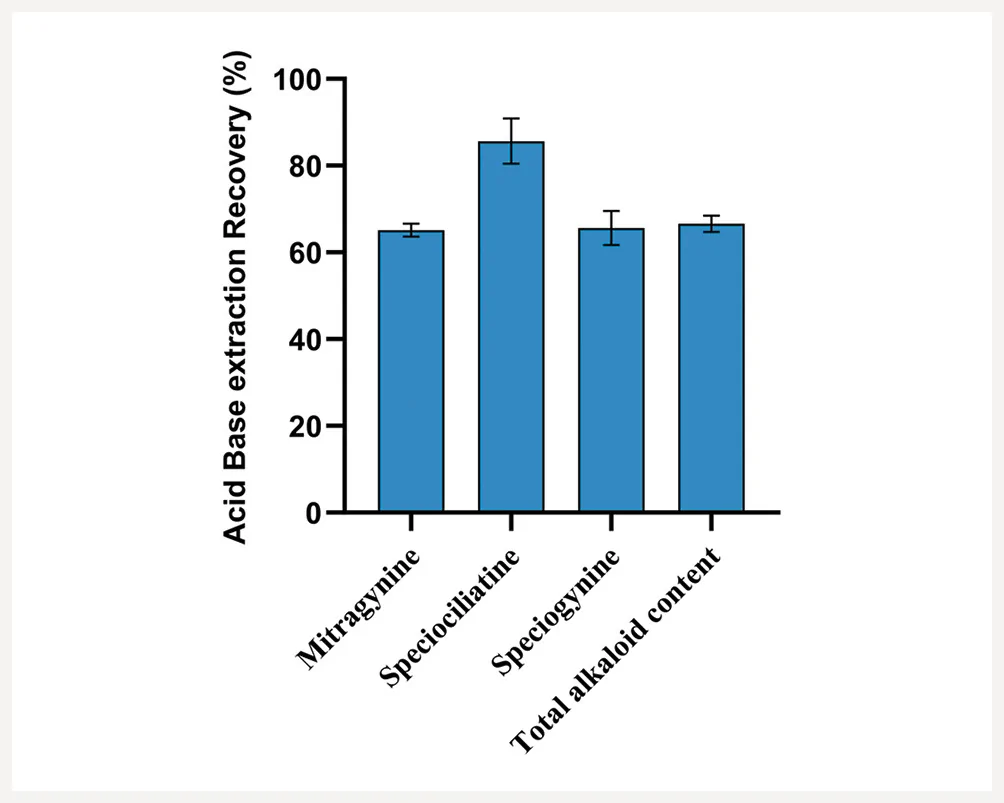
Recovery of mitragynine-type alkaloids from natural deep eutectic solvents (NaDES) extract by acid base extraction. Data are presented as mean ± SD (n = 6).

While this study demonstrates the high extraction efficiency of NaDES, a critical assessment of the overall process sustainability is necessary. A potential limitation of using NaDES is the requirement for downstream recovery steps (SPE, macroporous resins, or acid-base extraction) to isolate the alkaloids, which adds to the total process time and energy input. Compared to conventional organic solvents like methanol, which can be easily removed by simple evaporation, the recovery from NaDES involves additional reagents and materials. However, it should be noted that many conventional extractions also require subsequent purification to achieve similar levels of alkaloid enrichment. Furthermore, the use of food-grade NADES components significantly reduces the environmental and safety risks associated with toxic solvent disposal. While the additional recovery steps may partially offset the initial ‘greenʼ advantages in terms of complexity, the overall reduction in hazardous waste and the ability to reuse macroporous resins suggest that this method remains a more sustainable long-term alternative. Future studies should focus on life-cycle assessments (LCA) to quantify the precise energy and solvent consumption of the entire workflow compared to traditional methods.

Overall, this study demonstrates the potential of hydrophilic NaDES for extracting alkaloids from *M. speciosa* leaves. Forty-seven NaDES systems were screened, with extraction performance linked to properties such as viscosity and pH. The LaG NaDES showed the best extraction efficiency and was optimized to a molar ratio of HBA : HBD of 4 : 1 with 20% (w/w) water content. Additionally, key extraction parameters were optimized using a response surface methodology model. Target alkaloids were effectively recovered using solid phase extraction, macroporous resins, and acid-base extraction. Among the tested materials, Amberlite XAD 18 and Amberlite XAD 1600 N exhibited particularly high recovery rates across all targeted alkaloids. These findings highlight the effectiveness of NaDES systems as green solvents for the extraction and recovery of *Mitragyna speciosa* alkaloids and support its further investigation for practical applications. The significance of this optimization is underscored by the complex pharmacological profile of the plant; since alkaloids like mitragynine exhibit anti-inflammatory properties through cyclooxygenase-2 inhibition and interact with serotonin and calcium channels, a reproducible and ‘greenʼ protocol is essential for pharmaceutical standardization. By utilizing biocompatible, food-grade components, this study provides the necessary chemical framework to support the safe and effective therapeutic utilization of *M. speciosa* in clinical contexts.

## Materials and Methods

### Plant material

Dried *Mitragyna speciosa* (kratom) leaves were sourced from PT. Sepakat Multi Niaga (Indonesia) and authenticated morphologically by Dr. E. Kalpoutzakis (Affiliation, see Acknowledgments). A voucher specimen (NN112) was deposited at the Laboratory of Pharmacognosy, National and Kapodistrian University of Athens. Leaves were dried, milled to a uniform particle size and stored in airtight containers at room temperature until extraction.

### Chemicals and reagents

A kratom alkaloid mixture (mitragynine, mitraphylline, speciogynine, speciociliatine, and 7-hydroxy-mitragynine) was obtained from Cayman Chemicals. Chemicals for NaDES formulation, including choline chloride (ChCl, 98%), betaine (Be, 98%), proline (Pro, 99%), D-(−)-fructose (F, 99%), D-(+)-glucose (G, 99%), sucrose (S, 99%), citric acid (Ca, 99%), glycolic acid (Ga, 98%), DL-lactic acid (La, 80 – 85%), levulinic acid (Le, 98%), DL-malic acid (Ma, 98%), L-(+)-tartaric acid (Ta, 99%), and glycerol, were purchased from Alfa Aesar. Deionized water was obtained by a MILLI-Q system (Evoqua water technologies, LaboStar Pro TWF UV). Methanol, isopropyl alcohol, glacial acetic acid, acetone, and ammonia solution 25% were obtained from Merck (Merck KGaA). LC-MS-grade acetonitrile, LC-MS-grade methanol, LC-MS-grade formic acid, and ethanol absolute were procured from Fisher Chemical (Fisher Scientific).

### Liquid chromatography condition and method validation

Alkaloid quantification was performed on a Vanquish Duo UHPLC System (Thermo Fisher Scientific) coupled to a binary solvent manager, autosampler, heated column compartment, and photodiode array (PDA) detector and to an ISQ ^™^ EC Single Quadrupole Mass Spectrometer (Thermo Fisher Scientific). A gradient elution method was developed with solvent A (milli-Q water with 0.1% formic acid) and solvent B (acetonitrile). The linear gradient program was as follows: 0 min, 90% solvent A; 0 – 16 min, 50% solvent A and 16 – 17 min, 0% solvent A; 17 – 23 min, 0% solvent A; 23 – 24 min, 90% solvent A; and 24 – 30 min, 90% solvent A. Separation was achieved on a C18 (100 × 3 mm, 2.6 µm; Fortis Technologies Ltd.) column at 35 °C with a flow rate of 0.3 mL/min and an injection volume of 3 µL. Data acquisition and processing were performed using Chromeleon 7.3.2 (Thermo Fisher Scientific).

Quantification of alkaloids was carried out using PDA detection at 220 nm, while the mass spectrometer was used for compound confirmation. Representative chromatograms of the reference standards are provided in the Supplementary Information ( **Fig. 6S** ).

Calibration curves for each standard were constructed using serial dilutions of the alkaloid mixture (1 to 32 µg/mL), in which all standards were present at identical concentration, prepared in LC-MS grade methanol. Each calibration level was analyzed in triplicate. The linear regression equations were as follows:

y = 0.192 x + 0.07 665 (R ^2^ = 0.9991) for 7-hydroxy-mitragynine,

y = 0.1778 x + 0.0907 (R ^2^ = 0.9989) for mitraphylline,

y = 0.3929 x + 0.1811 (R ^2^ = 0.9990) for mitragynine,

y = 0.3997 x + 0.2079 (R ^2^ = 0.9989) for speciociliatine,

y = 0.4112 x + 0.1825 (R ^2^ = 0.9990) for speciogynine.

All the analytes showed significant linearity (R ^2^ > 0.998). The method was validated in terms of linearity (R ^2^ ), specificity (Rt), limit of detection (LOD), limit of quantification (LOQ), and precision (inter-day and intra-day) analysis ( **Tables 4S–6S** , Supporting Information).

### Screening process for the selection of the most efficient NaDES

NaDES of different molar ratios (1 : 1, 2 : 1, and 1 : 2 (mol _HBA_ : mol _HBD_ )) were freshly prepared by heating (80 ± 2 °C) with continuous stirring in a closed glass vial until a clear, homogeneous mixture was obtained 30 . Distilled water was added to a content 30% (v/v) of each mixture. In the current study, a total of 47 different NaDES solvent systems were prepared and evaluated ( **Table 1S** , Supporting Information).

The viscosity of each NaDES was measured using a glass viscometer (CFRC-300, CAN-FEN ROUTINE VISC CALIBRATED; CANNON instrument company) as previously described 19 .

The pH values of the NaDES solvent systems (containing 30% v/v water) were measured in triplicate at 25 ± 1 °C using a digital pH meter. The pH meter was calibrated using standard aqueous buffers at pH 4.0, 7.0, and 10.0 prior to analysis.

To select of the most efficient NaDES, microextraction of powdered *M. speciosa* leaves (100 mg) was performed using 1 mL of each NaDES. The mixture was vortexed for 1 min and then sonicated at 40 °C for 30 min in an ultrasound bath (Elmasonic S100 H; Elma Hans Schmidbauer GmbH & Co). The suspension was subsequently centrifuged at 2000 rpm for 10 min at 24 °C (MIKRO 220 R; Hettich GmbH & Co.KG). Then, 0.1 mL of the extract was diluted with 9.9 mL of LC-MS-grade methanol and filtered through a syringe PTFE filter (0.2 µm; Merck KGaA). For comparison, extractions using conventional solvents (methanol, Η _2_ Ο : AcOH 8 : 2, isopropyl alcohol, and acetone) were performed under identical conditions. All extractions were carried out in triplicate, and the data are presented as the average values with RSD < 5%.

### Extraction optimization

The most efficient NaDES was selected and optimized using a single-factor experiment by testing different molar ratios of HBA to HBD (1 : 1 to 5 : 1) and the varying water content of the NaDES system (0 – 40%), following the extraction protocol described above.

Optimization of the extraction parameters involved a Box–Behnken design with response surface methodology (RSM) to evaluate the effects of extraction time (A), extraction temperature (B), and solid-to-liquid ratio (C). A three-level (+ 1, 0, -1) Box–Behnken design was used to investigate the influence of these three independent variables on the extraction efficiency. Fifteen experimental runs, including three replications at the central point, were carried out in triplicate with the response being total alkaloids content. The average values of the triplicates were fitted with the following second-polynomial model (Eq. 1).

$Y=\;\beta_{0}\sum_{i=1}^{3}\beta_{i}\chi_{i}+\sum_{i=1}^{3}\beta_{ii}\chi_{ii}^{2}+\sum_{i=1}^{3}\sum_{j=i+1}^{3}\beta_{ij}{\chi_{i}\chi }_{j}$

where Y is the response variable, X _i_ and X _j_ are the independent variables of the model, and *β*_0_ , *β*_i_ , *β*_ii_ , and *β*_ij_ are the coefficient of intercept, linear, quadratic, and interactive coefficient, respectively. The experimental design and data analysis were performed with the software Design-expert 13.

### Alkaloid recovery from NaDES

Several recovery techniques were evaluated for *M. speciosa* alkaloids, including solid-phase extraction, macroporous resins, and acid-base extraction.

Silica-based cartridges, including C18 (Telos C18, 500 mg, 3 mL; Kinesis Scientific Experts) and HyperSep SCX (500 mg, 3 mL, Thermo Fisher Scientific), were used. A 1 mL aliquot of NaDES extract was loaded onto the cartridges. C18 cartridges were preconditioned with MeOH (10 mL) and equilibrated with H _2_ O (10 mL), followed by washes with H _2_ O (10 mL) and 20% EtOH abs/H _2_ O (10 mL) and elution with 10 mL EtOH abs. SCX cartridges were preconditioned with MeOH (10 mL) and equilibrated with H _2_ O/AcOH (8 : 2, 10 mL), washed with H _2_ O (10 mL) and MeOH (10 mL), and then eluted with 10 mL 5% NH _4_ OH in MeOH. Eluates were dried under nitrogen, redissolved in LC-MS methanol, and filtered before analysis through a syringe PTFE filter (0.2 µm; Merck KGaA) before analysis.

To recover the targeted compound, the polymeric absorbent resins Amberlite XAD-4, Amberlite XAD-HD7, Amberlite XAD-16 N, Amberlite XAD-1600 N, Amberlite XAD-1180 N, and Amberlite XAD-18 were evaluated. All the resins were purchased from Sigma-Aldrich. The characteristics of these polymeric adsorbent resins are summarized in **Table 7S** (Supporting Information). Prior to use, the resins were activated by washing with distilled water, then left overnight with ethanol (24 h), and finally washed again with distilled water. The recovery procedure was carried out using a 12 × 50 mm glass column packed with 2 mL resin. A total of 4 mL of extract was loaded onto the column at a flow rate of 0.2 mL/min. The column was then washed sequentially with distilled water (20 mL) and 15% ethanol (20 mL). Alkaloids were finally eluted with 20 mL of absolute ethanol at a flow rate of 2 mL/min. The collected eluates were dried, redissolved, and filtered as above.

A classic acid-base extraction approach was used to recover alkaloids from the NaDES extract 32 . An aliquot of 1 mL NaDES extract was diluted with 1 mL of water and basified to pH 8 – 9 using a 5% aqueous ammonia solution. The mixture was then extracted twice with 2 mL of chloroform by vortexing, followed by centrifugation at 4000 rpm for 10 min at 25 °C (5804 R, Eppendorf). The chloroform layer was collected, dried, redissolved, and filtered as above.

### Statistical analysis

Statistical significance was determined using one-way analysis of variance (ANOVA) followed by Tukeyʼs test for multiple comparisons, with a significance level set at p < 0.05. Correlation between physicochemical properties (viscosity and pH) and extraction yields was evaluated using Spearmanʼs rank correlation coefficient ( *ρ* ). The one-way ANOVA, Tukeyʼs test, and boxplot visualizations were conducted using GraphPad Prism 8.0.1. Scatter-plots with regression lines were generated in the open-source software RStudio 2025.5.0.496 with the package “ggplot2” 33 .

## Supporting Information

Physicochemical properties of NaDES solvent systems ( **Table 1S** ), experimental orders and response of the BBD method ( **Table 2S** ), ANOVA results of total alkaloid content model ( **Table 3S** ), method validation data ( **Tables 4S–6S** ), resin characteristics ( **Table 7S** ), extraction efficiency of NaDES solvent systems ( **Figs 1S–4S** ), and extraction efficiency of commonly used solvents ( **Fig 5S** ) are available as Supporting information.
Ergänzendes Material Supporting Information
