# Supplementary material for: Natural Deep Eutectic Solvent Extraction Optimization of Alkaloids from Mitragyna speciosa : A Methodological Evaluation
Source: Planta Med. 2026 Jun 11;92(12):1136–46. doi: 10.1055/a-2880-8498 (PMC13630439; doi:10.1055/a-2880-8498)
Supplement: Supplementary file 1 [file 10-1055-a-2880-8498-sup_pmk0201.pdf]

Supplementary material

Natural Deep Eutectic Solvent Extraction Optimization of Alkaloids from *Mitragyna speciosa*: A Methodological Evaluation

Christina Koufali[1], Alexandros Polyzois[1], Marina Kritsanida[2], Nektarios Aligiannis[1], Ioanna Chinou[1], Nikolas Fokialakis[1]

Affiliation

[1] Division of Pharmacognosy & Chemistry of Natural Products, Department of Pharmacy, School of Health Sciences, National and Kapodistrian University of Athens, Athens, Greece

[2] UMR CNRS 8038 CiTCom, Faculté de Pharmacie, Université Paris Cité Paris, France

Correspondence

Ass. Prof. Nikolas Fokialakis

Department of Pharmacy, National and Kapodistrian University of Athens,

Panepistimiopolis 15771 Athens, Greece

Phone: + 30 21 07 27 45 24

fokialakis@pharm.uoa.gr

Planta Med | DOI 10.1055/a-2880-8498 | © The Author(s).

Contents

Tables

Table 1S. Physicochemical properties of NaDES solvent systems. 3

Table 2S. Experimental orders and response of the BBD method. 4

Table 3S. ANOVA results of total alkaloid content model. 5

Table 4S. Linearity of the quantification method. 5

Table 5S. Specificity of the quantification method. 5

Table 6S. Precision of the quantification method. 5

Table 7S. Physical properties of different macro-porous resins used in this study. 6

Figures

Fig. 1S. Extraction efficiency of acid/sugar-based NaDES. 7

Fig. 2S. Extraction efficiency of choline-chloride-based NaDES. 7

Fig. 3S. Extraction efficiency of betaine-based NaDES. 8

Fig. 4S. Extraction efficiency of glycerol-based NaDES. 8

Fig. 5S. Extraction efficiency of commonly used solvents**.** **9**

Fig. 6S. Reference standard HPLC chromatogram of kratom alkaloids (7-hydroxy-mitragynine, mitraphylline, mitragynine, speciogynine, and speciociliatine) used for quantification recorded at 220 nm. **9**

Planta Med | DOI 10.1055/a-2880-8498 | © The Author(s).

Table 1S. Physicochemical properties of NaDES solvent systems.

HBA	HBD	NaDES abbr.	Ratio	Appearance	Viscosity (cP)	pH
Proline	D-(−)-Fructose	ProF	1:1	Clear yellow translucid liquid	138.30	6.54
Proline	D-(+)-Glucose	ProG	1:1	Clear light-yellow translucid liquid	223.20	6.2
Proline	Sucrose	ProS	1:1	Clear light-yellow translucid liquid	305.43	6.96
Proline	Glycerol	ProGly		Clear orange translucid liquid	30.25	6.88
Proline	Citric acid	ProCa	1:1	Clear translucid liquid	255.96	2.53
Proline	DL-Lactic acid	ProLa	1:1	Clear translucid liquid	27.69	2.64
Proline	Levulinic acid	ProLev	1:1	Clear light-yellow translucid liquid	22.60	4.05
Proline	DL-Malic acid	ProMa	1:1	Clear translucid liquid	114.58	2.84
Proline	L-(+)-Tartaric acid	ProTa	1:1	Crystals formation	nd	nd
Choline chloride	D-(−)-Fructose	ChclF	1:1	Clear translucid liquid	35.10	3.51
Choline chloride	D-(+)-Glucose	ChclG	1:1	Clear translucid liquid	44.60	2.53
Choline chloride	Sucrose	ChclS	1:1	Clear translucid liquid	54.27	6.61
Choline chloride	Glycerol	ChclGly	1:2	Clear translucid liquid	12.98	4.7
Choline chloride	Citric acid	ChclCa	1:1	Clear translucid liquid	41.45	2.56
Choline chloride	DL-Lactic acid	ChclLa	1:1	Clear translucid liquid	6.76	1.36
Choline chloride	Levulinic acid	ChclLev	1:1	Clear translucid liquid	8.92	2.12
Choline chloride	DL-Malic acid	ChclMa	1:1	Clear translucid liquid	21.85	0.77
Choline chloride	L-(+)-Tartaric acid	ChclTa	1:1	Clear translucid liquid	31.79	0.89
Betaine	D-(−)-Fructose	BeF	1:1	Clear translucid liquid	232.11	6.33
Betaine	D-(+)-Glucose	BeG	1:1	Clear translucid liquid	128.77	6.01
Betaine	Sucrose	BeS	1:1	Clear translucid liquid	207.85	7.52
Betaine	Glycerol	BeGly	1:2	Clear translucid liquid	24.03	5.54
Betaine	Citric acid	BeGly	1:1	Clear translucid liquid	92.28	3.48
Betaine	DL-Lactic acid	BeLa	1:1	Clear translucid liquid	14.94	3.85
Betaine	Levulinic acid	BeLa	1:1	Clear translucid liquid	20.79	4.45
Betaine	DL-Malic acid	BeMa	1:1	Clear translucid liquid	89.11	3.19
Betaine	L-(+)-Tartaric acid	BeTa	1:1	Clear translucid liquid	76.70	3.33
Citric acid	D-(−)-Fructose	CaF	2:1	Clear orange translucid liquid	210.18	0.83
Citric acid	D-(+)-Glucose	CaG	2:1	Clear translucid liquid	197.38	0.85
Citric acid	Sucrose	CaS	2:1	Clear yellow translucid liquid	229.07	0.88
Citric acid	Glycerol	CaGly	1:2	Clear orange translucid liquid	30.97	1.31
DL-Lactic acid	D-(−)-Fructose	LaF	2:1	Clear translucid liquid	10.70	1.92
DL-Lactic acid	D-(+)-Glucose	LaG	2:1	Clear translucid liquid	11.98	1.41
DL-Lactic acid	Sucrose	LaS	2:1	Clear translucid liquid	13.16	1.54
DL-Lactic acid	Glycerol	LaGly	1:2	Clear translucid liquid	11.34	1.77
Levulinic acid	D-(−)-Fructose	LevF	2:1	Clear translucid liquid	13.10	1.9
Levulinic acid	D-(+)-Glucose	LevG	2:1	Clear translucid liquid	14.89	1.86
Levulinic acid	Sucrose	LevS	2:1	Clear translucid liquid	16.67	2.03
Levulinic acid	Glycerol	LevGly	1:2	Clear translucid liquid	12.77	2.17
DL-Malic acid	D-(−)-Fructose	MaF	2:1	Clear light-yellow translucid liquid	64.86	1.15
DL-Malic acid	D-(+)-Glucose	MaG	2:1	Clear translucid liquid	79.21	1.03
DL-Malic acid	Sucrose	MaS	2:1	Clear light-yellow translucid liquid	74.67	1.33
DL-Malic acid	Glycerol	MaGly	1:2	Clear translucid liquid	12.12	1.35
L-(+)-Tartaric acid	D-(−)-Fructose	TaF	2:1	Clear yellow translucid liquid	151.23	0.9
L-(+)-Tartaric acid	D-(+)-Glucose	TaG	2:1	Clear translucid liquid	164.75	0.88

Planta Med | DOI 10.1055/a-2880-8498 | © The Author(s).

L-(+)-Tartaric acid	Sucrose	TaS	2:1	Clear yellow translucid liquid	23.63	1.08
L-(+)-Tartaric acid	Glycerol	TaGly	1:2	Clear light-yellow translucid liquid	29.80	1.34

Table 2S. Experimental orders and response of the BBD method.

Runs	Extraction temperature (°C)	Extraction time (min)	Solid-to-liquid ratio (mg/ml)	Total alkaloi content (mg/g DW)
1	50	40	80	26.05
2	50	10	30	27.35
3	30	10	80	25.51
4	50	40	80	25.93
5	50	10	130	23.65
6	50	40	80	25.78
7	70	40	30	27.65
8	70	70	80	25.95
9	30	70	80	25.65
10	50	70	130	23.45
11	30	40	130	23.28
12	70	10	80	26.95
13	50	70	30	27.57
14	70	40	130	23.78
15	30	40	30	27.52

Planta Med | DOI 10.1055/a-2880-8498 | © The Author(s).

Table 3S. ANOVA results of total alkaloid content model.

Source	Sum of Squares	df	Mean squares	F-values	p-value	
Model	33.61	9	3.73	64.04	0.0001	significant
Residual	0.2916	5	0.0583			
Lack of fit	0.2550	3	0.0850	4.64	0.1822	not significant
Pure error	0.0366	2	0.0183			
R^2	0.9759					

Table 4S. Linearity of the quantification method.

Standard abbr.	Mean peak area at different concentrations ± SD (n = 9)					
	**1 µg/ml**	**2 µg/ml**	**4 µg/ml**	**8 µg/ml**	**16 µg/ml**	**32 µg/ml**
**7-hydroxy-mitragynine**	0.22 ± 0.02	0.42 ± 0.01	0.83 ± 0.02	1.66 ± 0.04	3.26 ± 0.052	6.15 ± 0.134
**Mitraphyllinne**	0.20 ± 0.01	0.41 ± 0.01	0.79 ± 0.01	1.58 ± 0.04	3.06 ± 0.064	5.71 ± 0.116
**Mitragynine**	0.44 ± 0.02	0.89 ± 0.03	1.75 ± 0.05	3.44 ± 0.103	6.71 ± 0.105	12.61 ± 0.283
**Speciociliatine**	0.45 ± 0.02	0.91 ± 0.03	1.82 ± 0.05	3.52 ± 0.104	6.89 ± 0.135	12.83 ± 0.265
**Speciogynine**	0.46 ± 0.02	0.92 ± 0.02	1.82 ± 0.04	3.59 ± 0.092	7.01 ± 0.128	13.19 ± 0.298

Table 5S. Specificity of the quantification method.

Specificity			
**Standard abbr.**	**Retention time**	**LOD (µg/ml)**	**LOQ (µg/ml)**
7-hydroxy- mitragynine	8.623 ± 0.055	0.284	0.947
Mitraphyllinne	9.236 ± 0.059	0.254	0.845
Mitragynine	13.274 ± 0.090	0.065	0.216
Speciociliatine	13.751 ± 0.092	0.044	0.145
Speciogynine	14.261 ± 0.094	0.069	0.229

Table 6S. Precision of the quantification method.

Standard abbr.	Intra-day precision (RSD, %) n=3	Inter-day precision (RSD, %) n=9
7-hydroxy- mitragynine	0.134	0.654
Mitraphyllinne	0.232	0.745
Mitragynine	0.321	0.896
Speciociliatine	0.145	0.643
Speciogynine	0.435	0.756

Planta Med | DOI 10.1055/a-2880-8498 | © The Author(s).

Table 7S. Physical properties of different macro-porous resins used in this study.

Resin type	Matrix active group	Particle size	Surface area (m^2/g)	Average pore size (Å)
Amberlite XAD 7HP	Crosslinked aliphatic polymer (acrylic)	560–710 µm	≥ 380	300–400
Amberlite XAD 4	Crosslinked aromatic polymer (styrene–divinylbenzene)	490–690 µm	≥ 750	100
Amberlite XAD-16N	Crosslinked aromatic polymer (styrene–divinylbenzene)	560–710 µm	≥ 800	200
Amberlite XAD-1600N	Crosslinked aromatic polymer (styrene–divinylbenzene)	400–500 µm	≥ 800	200
Amberlite XAD-16HPN	Crosslinked aromatic polymer (styrene–divinylbenzene)	600–750 µm	≥ 800	800
Amberlite XAD-18	Crosslinked aromatic polymer (styrene–divinylbenzene)	400–500 µm	≥700	150
Amberlite XAD-1180N	Crosslinked aromatic polymer (styrene–divinylbenzene)	350–600 µm	≥600	400

Planta Med | DOI 10.1055/a-2880-8498 | © The Author(s).

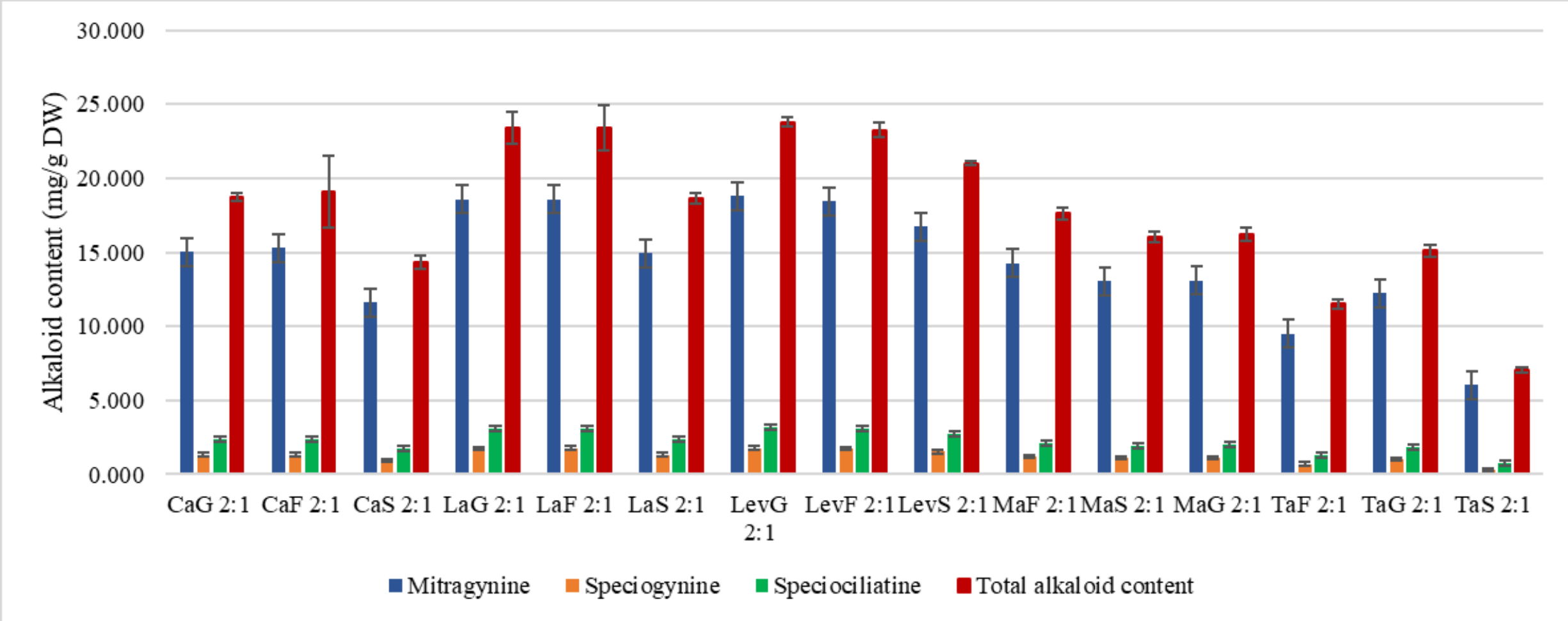

Fig. 1S. Extraction efficiency of acid/sugar-based NaDES.

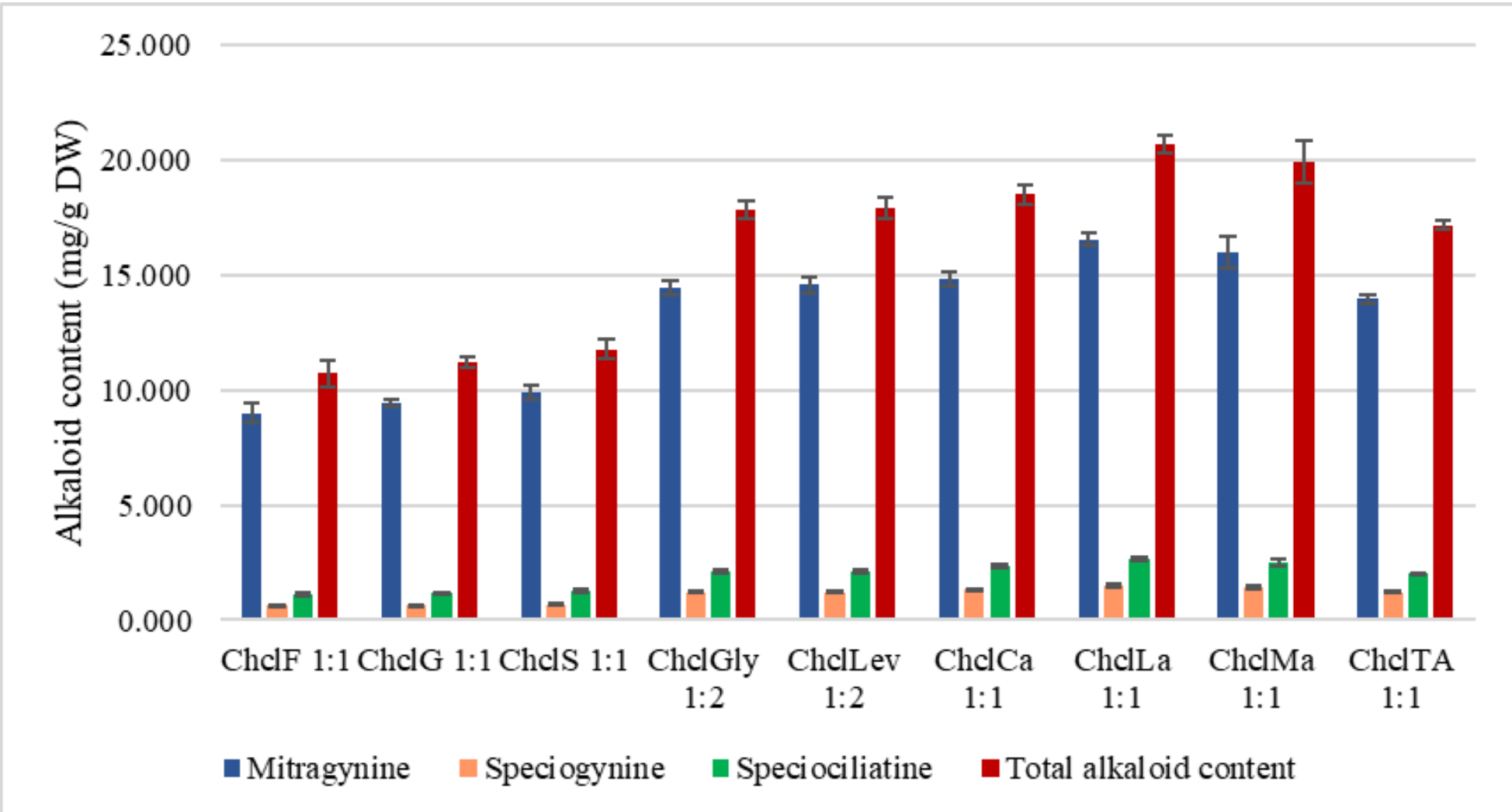

Fig. 2S. Extraction efficiency of choline-chloride-based NaDES.

Planta Med | DOI 10.1055/a-2880-8498 | © The Author(s).

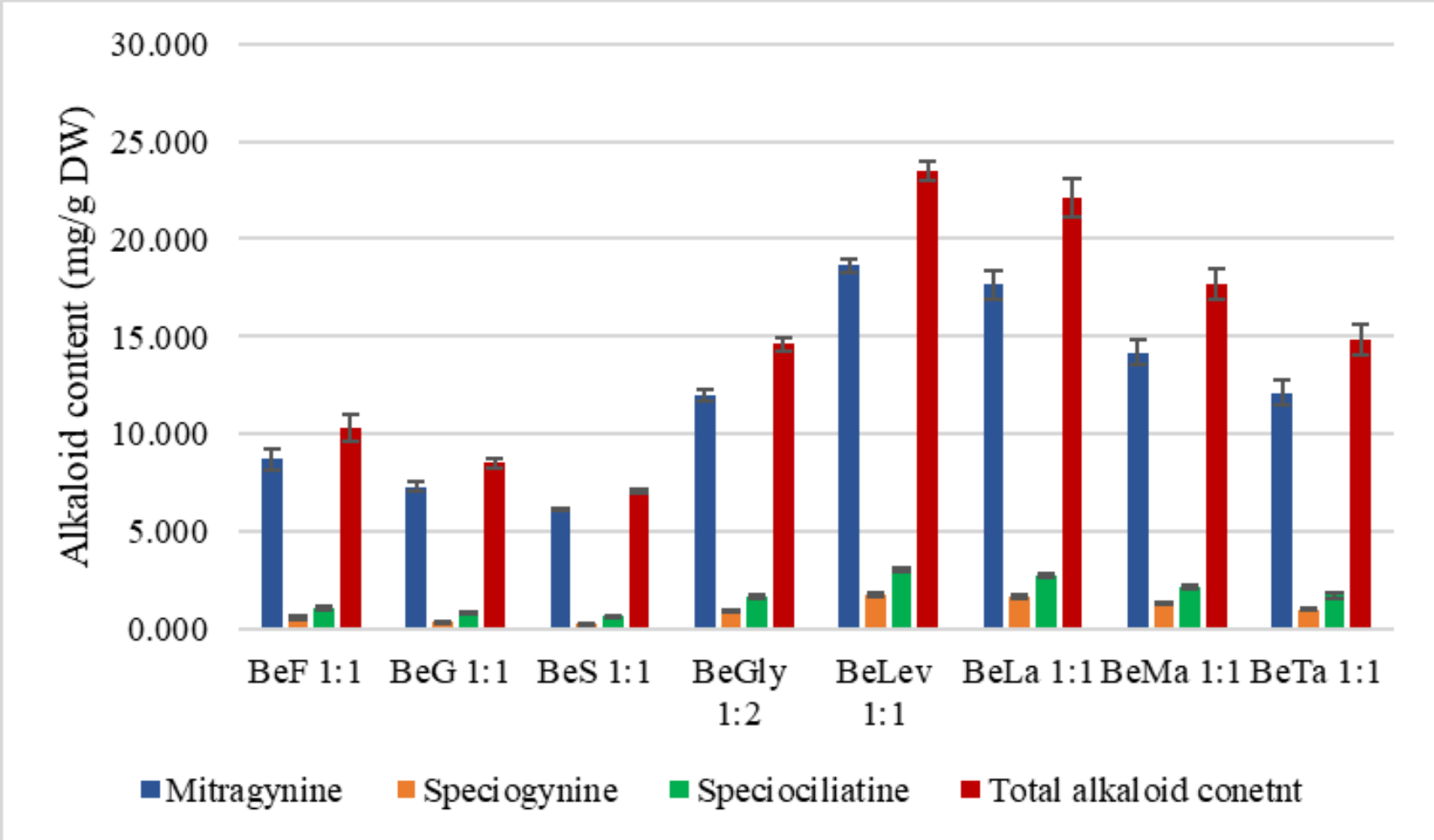

Fig. 3S. Extraction efficiency of betaine-based NaDES.

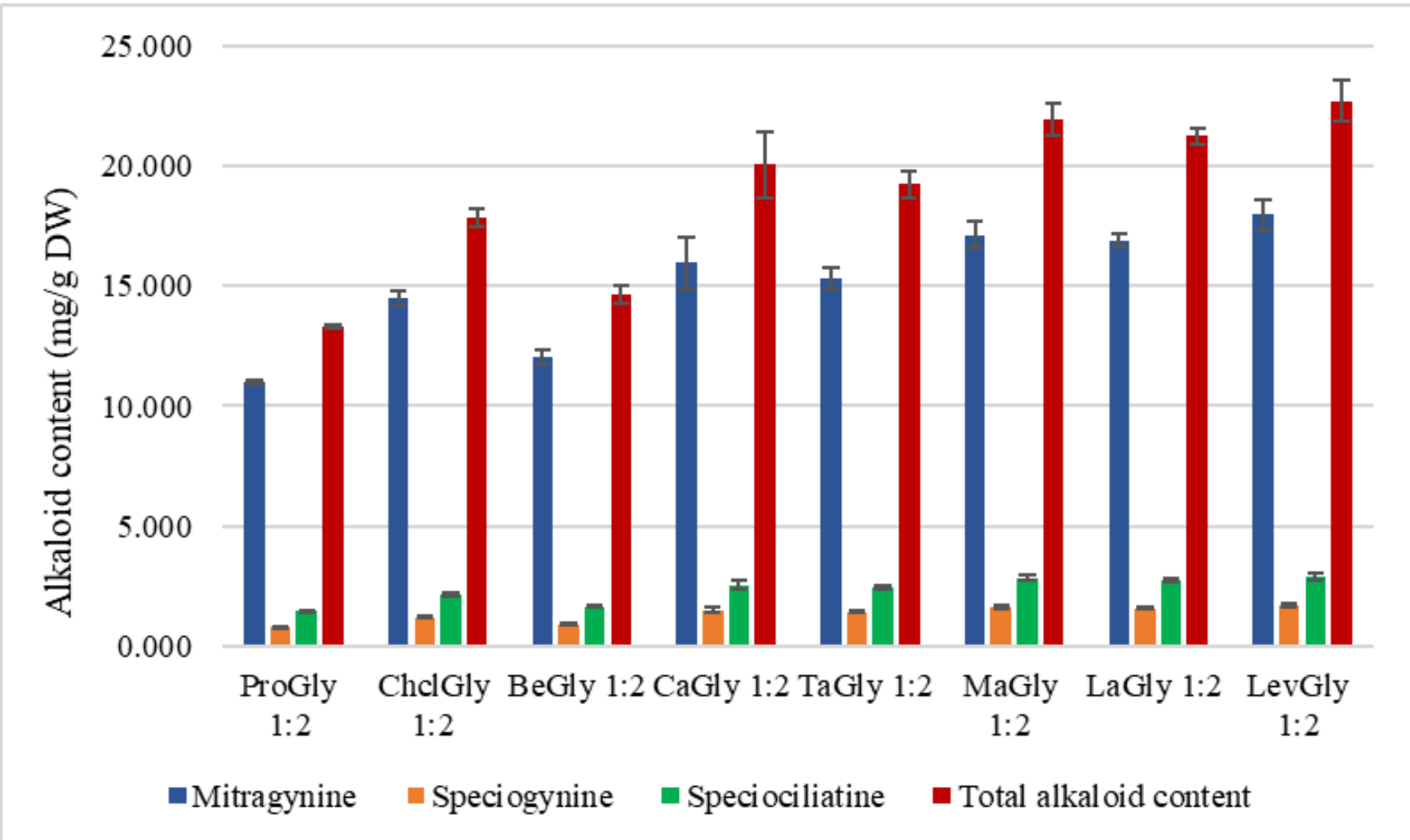

Fig. 4S. Extraction efficiency of glycerol-based NaDES.

Planta Med | DOI 10.1055/a-2880-8498 | © The Author(s).

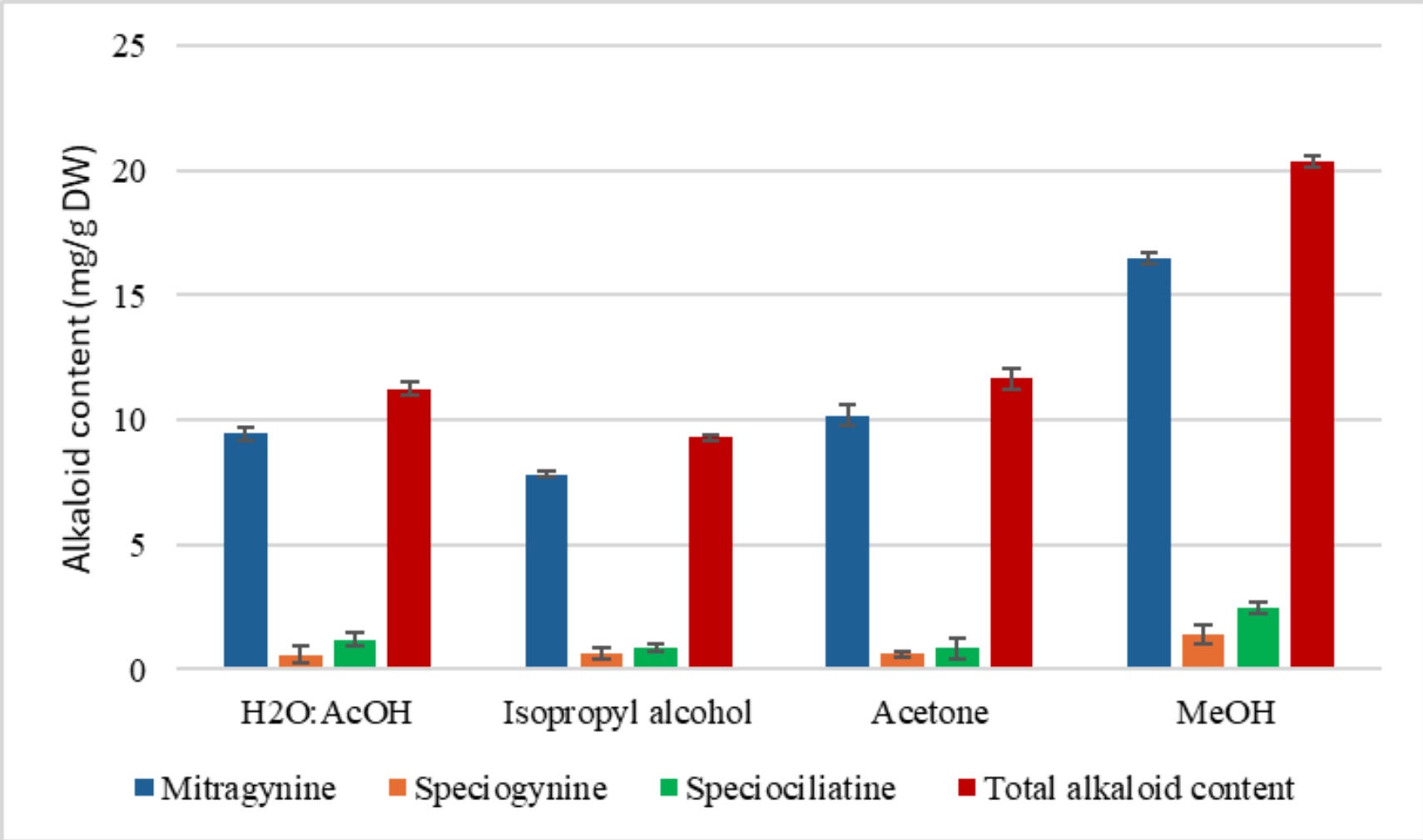

Fig. 5S. Extraction efficiency of commonly used solvents**.**

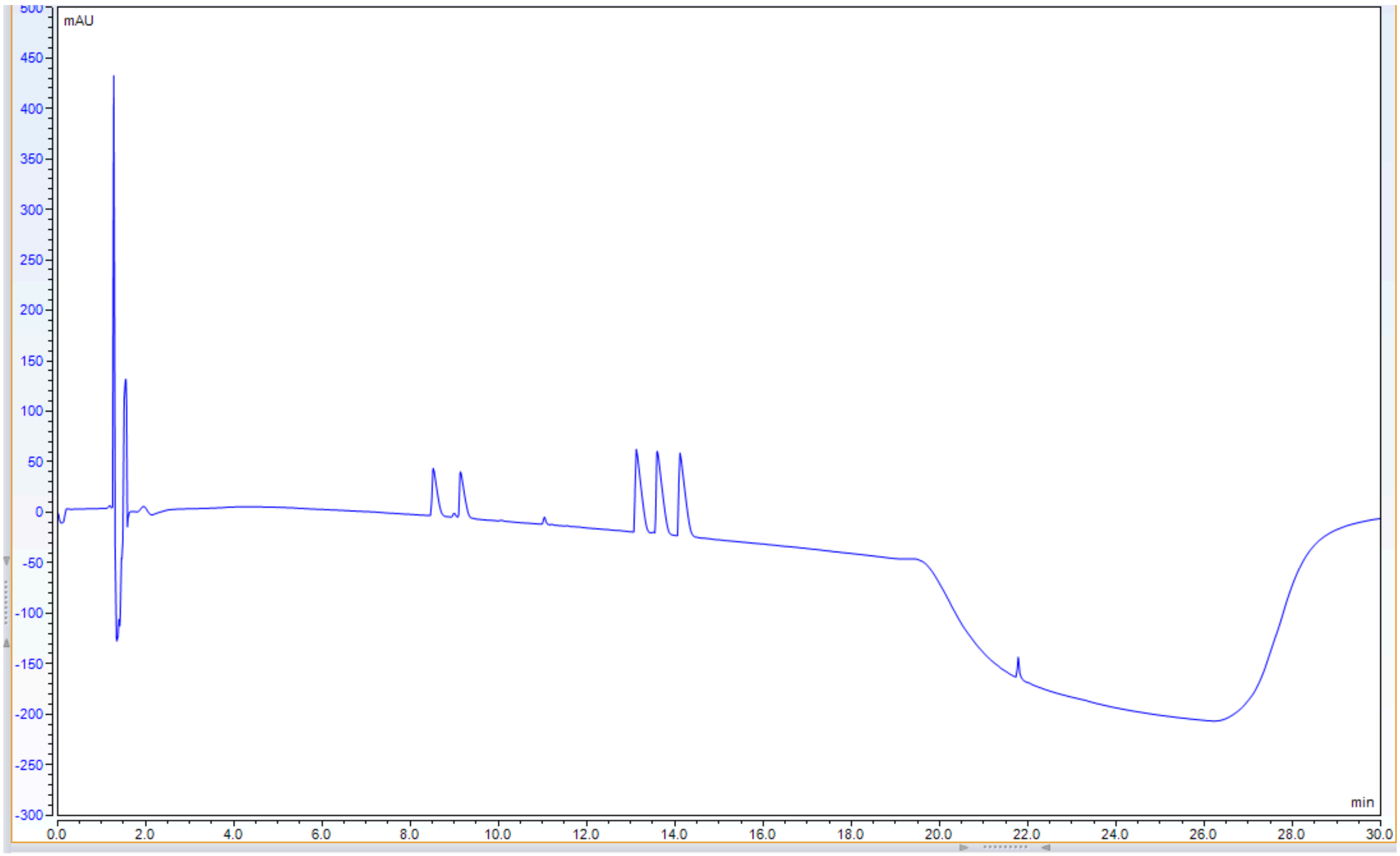

Fig. 6S. Reference standard HPLC chromatogram of kratom alkaloids recorded at 220 nm and used for quantification. Peaks, in order of elution, correspond to the following: (1) 7-hydroxy-mitragynine, (2) mitraphylline, (3) mitragynine, (4) speciogynine, and (5) speciociliatine.

Planta Med | DOI 10.1055/a-2880-8498 | © The Author(s).
